# Segmenting Patients With Diabetes With the Navigator Service in Primary Care and a Description of the Self-Acting Patient Group: Cross-Sectional Study

**DOI:** 10.2196/40560

**Published:** 2023-09-08

**Authors:** Riikka Riihimies, Elise Kosunen, Tuomas H Koskela

**Affiliations:** 1 Faculty of Medicine and Health Technology Tampere University Tampere Finland; 2 Center of General Practice Tampere University Hospital Tampere Finland

**Keywords:** patient segmentation, Navigator, self-acting patient, diabetes, primary care, self-management, skills, care, nurse, medication, quality of life, well-being, digital, patient

## Abstract

**Background:**

The aim of patient segmentation is to recognize patients with similar health care needs. The Finnish patient segmentation service Navigator segregates patients into 4 groups, including a self-acting group, who presumably manages their everyday life and coordinates their health care. Digital services could support their self-care. Knowledge on self-acting patients’ characteristics is lacking.

**Objective:**

The study aims are to describe how Navigator assigns patients with diabetes to the 4 groups at nurses’ appointments at a health center, the self-acting patient group’s characteristics compared with other patient groups, and the concordance between the nurse’s evaluation of the patient’s group and the actual group assigned by Navigator (criterion validity).

**Methods:**

Patients with diabetes ≥18 years old visiting primary care were invited to participate in this cross-sectional study. Patients with disability preventing informed consent for participation were excluded. Nurses estimated the patients’ upcoming group results before the appointment. We describe the concordance (%) between the evaluation and actual groups. Nurses used Navigator patients with diabetes (n=304) at their annual follow-up visits. The self-acting patients’ diabetes care values (glycated hemoglobin [HbA1c], urine albumin to creatinine ratio, low-density lipoprotein cholesterol, blood pressure, BMI), chronic conditions, medication, smoking status, self-rated health, disability (World Health Organization Disability Assessment Schedule [WHODAS] 2.0), health-related quality of life (EQ-5D-5L), and well-being (Well-being Questionnaire [WBQ-12]) and the patients’ responses to Navigator’s question concerning their digital skills as outcome variables were compared with those of the other patients. We used descriptive statistics for the patients’ distribution into the 4 groups and demographic data. We used the Mann-Whitney U test with nonnormally distributed variables, independent samples *t* test with normally distributed variables, and Pearson chi-square tests with categorized variables to compare the groups.

**Results:**

Most patients (259/304, 85.2%) were in the self-acting group. Hypertension, hyperlipidemia, and joint ailments were the most prevalent comorbidities among all patients. Self-acting patients had less ischemic cardiac disease (*P*=.001), depression or anxiety (*P*=.03), asthma or chronic obstructive pulmonary disease (*P*<.001), long-term pain (*P*<.001), and related medication. Self-acting patients had better self-rated health (*P*<.001), functional ability (*P*<.001), health-related quality of life (*P*<.001), and general well-being (*P*<.001). All patients considered their skills at using electronic services to be good.

**Conclusions:**

The patients in the self-acting group had several comorbidities. However, their functional ability was not yet diminished compared with patients in the other groups. Therefore, to prevent diabetic complications and disabilities, support for patients’ self-management should be emphasized in their integrated care services. Digital services could be involved in the care of patients willing to use them. The study was performed in 1 health center, the participants were volunteers, and most patients were assigned to self-acting patient group. These facts limit the generalizability of our results.

**International Registered Report Identifier (IRRID):**

RR2-10.2196/20570

## Introduction

The digitalization of health care in recent years has been impressive. There are multiple digital alternatives for realizing health services, supporting patients’ care, and delivering care information to patients [1-5]. Digitalization increases accessibility in health care, and therefore, it may improve equity in delivering services to, for example, rural areas [3,6,7]. Additionally, digital interventions may efficiently and safely improve patients’ self-management skills [1,3-6,8-10].

However, digital services are not appropriate for all patients. Some patients may lack adequate Internet access, digital skills, or the capability to utilize information from digital devices [11-15]. Barriers to adopting digital services are related to old age, ethnicity, lower education and socioeconomic status, and disability [11,12]. Patients have concerns about losing their relationship with professionals or “being lost in the data” [11,14]. This digital divide and the risk of digital inequalities [15-18] must be noted when considering what kinds of patient groups to target with digital services.

Population or patient segmentation methods aim to recognize homogenous patient groups with similar needs in health care services in order to tailor and target appropriate, cost-effective, and medically effective services to these groups [19-21]. Data-driven or expert-driven segmentation methods usually manage large data sets and multiple variables [19-21], thus disregarding the individuality of patients. Therefore, these methods should be supplemented with methods that additionally consider patients’ views of their self-management and coping in everyday life, their digital skills, and their preferences for using digital health services.

**Figure 1:**
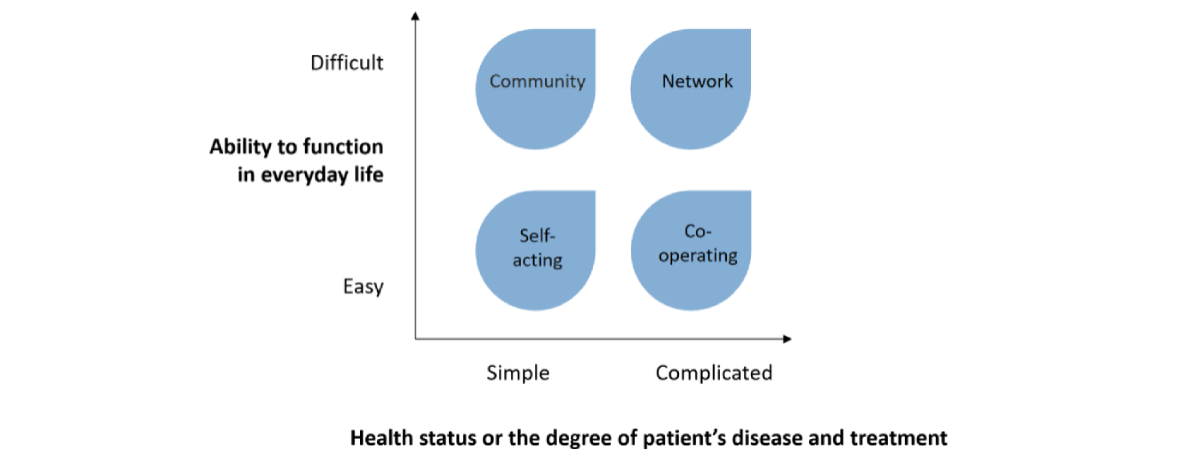
The 4 groups determined by Navigator: The functional ability in everyday life is studied with questions for patients, and the health status or the degree of the patient’s disease and treatment is determined using questions for health care professionals [22]. Adapted from Koivuniemi et al [23] with permission from Kustannus Oy Duodecim.

Navigator (*Suuntima* in Finnish) is a digital, nonprofit patient segmentation service developed in Finland. The service segregates patients into 4 groups (Figure 1), and each group has a separate care pathway. The pathways advise health care professionals about coordinating patients’ health services and advise patients about utilizing the most appropriate services. However, the group result does not affect the patient’s medical care. Navigator is based on questions for both professionals and patients. Patients are queried on the capability to manage in everyday life, and professionals are queried on the patient’s health status and complexity of care. The development of the Navigator service within the Kurkiaura project, patients’ and professionals’ questions and response options on a visual analog scale (VAS), description of the 4 groups, and proposed care pathways have been described in Navigator’s validation study protocol article by Riihimies et al [22].

One of the groups Navigator proposes is the “self-acting group.” Professionals have evaluated these patients’ health state and care as simple, and patients themselves have evaluated their coping and resources in everyday life as good or strong. Therefore, the patients are presumed to competently manage their everyday life and independently coordinate their health care. Health care services aim to support patients’ self-care in maintaining the ability to work and function. The individual health care plan for these patients focuses on self-care and supporting self-management. These patients could benefit from digital services (eg, for self-monitoring their care and contacting health care professionals). Remote appointments or contacts could be an appropriate alternative to health center visits [22].

Navigator is a generic service; therefore, it is suitable to be used with not only patients with diabetes but also any patients with chronic conditions. However, we chose patients with diabetes as the study population as diabetes is a major, potentially serious, and expensive chronic condition that induces multiorgan complications throughout the world [24]. Segmenting this vast population and offering targeted and appropriate services for different groups of patients with diabetes help focus care resources equally, thus improving all patients’ care results. From the perspective of the health care system, these actions could additionally reduce the costs of care.

Knowledge on the characteristics of the self-acting patient group and their ability to use digital services is currently lacking. We hypothesized that self-acting patients are younger and their medical condition is simpler compared with patients in the other groups Navigator proposes. In addition, we hypothesized that the self-acting patients’ self-rated health, functional ability, health-related quality of life and well-being, and digital skills are better than those in the other groups.

This is the first study concerning the characteristics of self-acting patients with diabetes formed by Navigator in a primary health care setting. In addition, the criterion validity examined here is one section of Navigator’s overall validation study. Criterion validity in general means evaluating a new instrument in comparison with the previously used “gold standard” [25]. In the case of Navigator, this is the professional’s evaluation of the patient’s group result based on previous knowledge of the patient or electronic health records (EHRs).

The aim of this study was to describe how Navigator assigns patients with diabetes to the 4 groups and the characteristics of the self-acting patient group. Further, we assessed the criterion validity of the Navigator service.

The detailed study questions are as follows:

1. How are patients with diabetes assigned to Navigator’s 4 groups at nurses’ appointments at a health center?
2. What kind of patients are assigned to the self-acting group, and how do the self-acting patients compare with other patients in terms of age, diabetes care values and medication, multimorbidity and medication, self-rated health, disability, health-related quality of life and well-being, and digital skills?
3. What is the agreement between the nurse’s estimate of the patient’s Navigator group result based on the care relationship or EHRs and the actual Navigator result (criterion validity)?

## Methods

### Study Design and Setting

This was an observational cross-sectional study. The detailed patient recruitment and data collection methods, the study process with patients with diabetes and nurse professionals at the health center, and the questionnaires’ contents are described in Navigator’s validation study protocol [22]. The data collection was accomplished between October 2018 and September 2019.

### Participants

Adult (≥18 years of age) patients with diabetes scheduled for a nurse’s annual control appointment were recruited for the study, and the patients volunteering to participate provided informed consent. Patients with a disability preventing informed consent for participation (eg, Alzheimer disease, mental disability) were excluded.

### Data Collection

#### Measurements and Variables

##### Navigator Database Information

The nurses used the Navigator service with patients with diabetes at their annual appointments at a health center. The Navigator database information was provided during data collection. In this study, we used the group distribution result. To measure patients’ digital skills, we additionally analyzed the patients’ responses to Navigator’s question “Do you know how to use electronic services?” Patients responded to this question on a VAS. The response options and values at its ends were “Yes” (=1) or “No” (=10).

##### Diabetes Care Values

We collected the patients’ diabetes care values for glycated hemoglobin (HbA_1c_), urine albumin to creatinine ratio (UACR), low-density lipoprotein (LDL) cholesterol, blood pressure, and BMI from the EHR.

##### Questionnaires

We used questionnaires that the nurses delivered to the patients at the appointments. Patients responded to the questionnaires after the appointment and returned them to the health center office in person or by mail.

A self-generated questionnaire assessed the variables of the patients’ chronic conditions and medication, smoking status, and self-rated health [26]. The World Health Organization Disability Assessment Schedule (WHODAS) 2.0 [27], a health-related quality of life measure (EQ-5D-5L) [28], and the Well-being Questionnaire (WBQ-12) [29] were used as well.

Self-rated health was measured with the question “How satisfied are you with your health?” on a scale from 1 (very dissatisfied) to 5 (very satisfied) [26]. We combined responses of 1 with 2 and 4 with 5, forming 3 response categories (1=unsatisfied, 2=neither satisfied nor unsatisfied, 3=satisfied).

The WHODAS 2.0 12-item questionnaire measures 6 domains of function (cognition, mobility, self-care, getting along, life activities, and participation) [27]. We analyzed the questionnaire with a simple scoring method, with which the patient’s responses to the 12 questions on 5 levels (from 1 “no difficulties” to 5 “extreme difficulties or could not”) were summed, formulating a result score from 12 to 60.

The 5 questions in the EQ-5D-5L represent the 5 dimensions of health (mobility, self-care, usual activities, pain or discomfort, and anxiety or depression). Patients respond to the questionnaire on 5 response levels (from “no problems” to “extreme problems or unable to”) [28]. The Euroqol EQ-5D-5L Crosswalk Index calculator was used to analyze the responses. To calculate the EQ-5D-5L index values, we used the Danish value set, as a Finnish value set does not exist and Denmark most closely approximates Finland. The Danish EQ-5D-5L formulates an index between 1 and –0.757 depicting health-related quality of life. An index score of 1 means perfect health, and 0 means death; negative index scores mean states considered worse than death [30]. We also analyzed the patients’ evaluation of their current health on an EQ VAS, on which 0 means “The worst health you can imagine” and 100 means “The best health you can imagine” [28].

The WBQ-12 dimensions of negative well-being, energy, positive well-being, and general well-being were calculated as guided by the licensee of the questionnaire (Health Psychology Research). Patients evaluated their well-being on a 4-point scale from 0 (not at all) to 3 (all the time) [29].

We compared the self-acting patients' outcomes with cooperation and network patients’ outcomes with respect to medical condition. Due to the small group sizes, the cooperation and network groups were merged in the statistical analysis. Additionally, the self-acting patients’ outcomes were compared with all other patients’ outcomes with respect to self-rated health, disability, health-related quality of life, and well-being. Due to the small group sizes, these 3 groups (cooperation, community, and network groups) were merged in the statistical analysis.

#### Criterion Validity

Nurses estimated the participating patients’ upcoming Navigator group results based on the EHR or previous care relationship before the appointment. This estimation was compared with the actual Navigator result to assess Navigator’s criterion validity.

### Study Size

The sample size and power calculation were based on results of a 36-item WHODAS 2.0 validation study of patients with chronic conditions in Europe. The calculation used a power of 80% and statistical significance of *P*=.05. The result of the calculation was a total sample size of 300 patients. The details of the sample size calculation are presented in our Navigator study protocol [22].

### Statistical Analysis

Patients’ characteristics and their distribution into the different groups by gender and age groups are described with frequencies and proportions. The Kruskal-Wallis test was used to compare the patients’ median age in the different groups [31,32]. The concordance and mismatch between the nurse’s evaluation of Navigator’s result and actual result are also described with proportions.

The Pearson chi-square test was used with categorized variables to compare the self-acting and other patient groups’ characteristics, smoking, chronic conditions and medication, and self-rated health. The distribution of scale variables (age distribution, years since the diabetes diagnosis, diabetes care values, WHODAS 2.0, EQ-5D-5L VAS, WBQ-12, and Navigator’s question “Do you know how to use electronic services?” on a VAS) were explored with the Kolmogorov-Smirnov test. For normally distributed variables, the independent samples *t* test was used to compare variable means, whereas the Mann-Whitney *U* test was used to compare medians if the variable was not normally distributed.

We describe frequencies of missing values that were excluded from the analyses. We analyzed the data with SPSS versions 25 and 26 (IBM Corp).

### Ethical Considerations

Tampere University Hospital’s Ethics Committee approved this study’s ethical aspects in October 2018 (ETL R18070). Data collection at Valkeakoski Health Center was approved by the head physician in September 2018. All patients and nurses participating in the study were provided with an informed consent form detailing the study aims and process, their voluntary participation, and ability to end participation without any consequences. We anonymized all data; thus, the data were examined without identifying information. We did not provide any compensation to the study participants.

## Results

### Participant Characteristics

Altogether, 538 patients were invited to the study, and 304 (56.5%) participated. Most participants (272/304, 89.5%) returned the study questionnaires. Nurses completed Navigator’s questionnaires with all 304 patients during an appointment. The majority of patients (232/304, 76.3%) were 60 years to 79 years old, and their gender distribution was balanced (Table 1).

Navigator segregated patients into the 4 groups, as follows**:** 259 into the self-acting group, 34 into the cooperating group, 6 into the community group, and 5 into the network group (Table 2).

In the self-acting group, the majority of patients (206/259, 79.6%) were 60 years to 79 years old, and their gender distribution was balanced. Patients were mostly married or in a relationship (160/236, 67.8%), and their school education was comprehensive (82/235, 34.9%) or vocational (93/235, 39.6%). The majority (199/236, 84.3%) were retired. Self-acting patients’ median age did not significantly differ from other patients’ median age; however, the proportion of patients ≥80 years old was smaller in the self-acting patient group (Multimedia Appendix 1, Table 3).

**Table 1:** Baseline characteristics of all patients with diabetes (n=304) participating in the study.

Characteristic			Value
Age (years), mean (SD)			68.9 (8.97)
Age (years), range			30-90
**Age range (years), n (%)**			
	≤59	40 (13.2)	
	60-69	109 (35.9)	
	70-79	123 (40.5)	
	≥80	32 (10.5)	
**Gender, n (%)**			
	Female	156 (51.3)	
	Male	148 (48.7)	
**Marital status, n (%)^a^**			
	Unmarried	9 (3.3)	
	Married (in relationship)	178 (65.9)	
	Divorced	40 (14.8)	
	Widowed	43 (15.9)	
**School education, n (%)^a^**			
	Comprehensive	101 (37.4)	
	Secondary school graduate	4 (1.5)	
	Vocational	102 (37.8)	
	College	44 (16.3)	
	Academic	19 (7.0)	
**Employment, n (%)^b^**			
	Employed (including self-employment)	28 (10.3)	
	Unemployed	11 (4.1)	
	Unable to work	3 (1.1)	
	Retired	229 (84.5)	

^a^34 missing values.

^b^33 missing values.

**Table 2:** Patients’ (n=304) distribution into Navigator’s 4 groups (self-acting, cooperating, community, and network) and gender distribution within each group.

Group	Total sample, n (%)	Women, n (%)	Men, n (%)
Self-acting	259 (85.2)	133 (51.4)	126 (48.6)
Cooperating	34 (11.2)	19 (55.9)	15 (44.1)
Community	6 (2)	2 (33.3)	4 (66.7)
Network	5 (1.6)	2 (40)	3 (60)

**Table 3:** Comparison of self-acting patient group (n=259) and all other patient groups (n=45) according to age groups.

Group	Age groups (years), n (% within group)					Total sample, n (%)^a^		Q1^b^		Median^c^		Q3^d^		SD
	≤59	60-69	70-79	≥80										
Self-acting	32 (12.4)	97 (37.5)	109 (42.1)	21 (8.1)	259 (85.2)		65		70		74		8.4	
All other groups	8 (17.8)	12 (26.7)	14 (31.1)	11 (24.4)	45 (14.8)		62.5		71		79		11.6	

^a^*P*=.005 (calculated using crosstabulation in the Pearson chi-square test).

^b^Q1: lower quartile.

^c^*P*=.17 (calculated using the groups' age medians in the Mann-Whitney *U* test).

^d^Q3: upper quartile.

### Diabetes Care Values

The self-acting patients had lived with a diabetes diagnosis for less than 10 years, and the result differed from the cooperation and network groups. The difference was statistically significant (*P*=.003; Table 4).

The self-acting patients’ HbA_1c_ values differed from those of the cooperation and network groups, and the difference was statistically significant (*P*<.001), as was the difference in LDL cholesterol results between groups (*P*=.04). The differences in other diabetes care values (UACR, systolic and diastolic blood pressures, BMI) and smoking status between groups were not statistically significant (Table 4).

**Table 4:** The self-acting patient group’s (n=259) and the combined cooperation and network groups’ (n=39) diabetes care values.

Variable				Self-acting group			Cooperation and network groups			*P* value		
**Duration with diabetes mellitus (years)**												
	Mean (SD)	10.04 (8.60)			14.00 (8.51)			—^a^				
	Range	0-66			1-39			—				
	Q1^b^	5.00			9.50			—				
	Median	8.00			12.00			.003^c^				
	Q3^d^	13.00			20.00			—				
	Missing values, n	50			9			—				
**HbA_1c_ (mmol/mol)**												
	Mean (SD)	45.55 (9.49)			54.28 (11.73)			—				
	Range	31-116			38-83			—				
	Q1	39.00			34.00			—				
	Median	43.00			54.00			<.001^c^				
	Q3	50.00			63.00			—				
	Missing values, n	2			0			—				
**Low-density lipoprotein (LDL) cholesterol (nmol/L)**												
	Mean (SD)	2.40 (0.84)			2.27 (1.11)			—				
	Range	0.9-6.0			0.9-5.4			—				
	Q1	1.80			1.55			—				
	Median	2.3			1.8			.04^c^				
	Q3	2.80			2.80			—				
	Missing values, n	11			2			—				
**Urine albumin to creatinine ratio (mg/mmol)**												
	Mean (SD)	2.35 (7.07)			3.89 (11.48)			—				
	Range	0.2-62.9			0-57.2			—				
	Q1	0.40			0.50			—				
	Median	0.60			0.80			.25^c^				
	Q3	1.30			1.40			—				
	Missing values, n	23			4			—				
**Systolic blood pressure (mm Hg)**												
	Mean (SD)	134.33 (13.16)			137.08 (16.79)			—				
	Range	105-196			114-191			—				
	Q1	127.0			127.5			—				
	Median	135.0			136.0			.58^c^				
	Q3	141.0			143.5			—				
	Missing values, n	5			2			—				
**Diastolic blood pressure (mm Hg)**												
	Mean (SD)	78.04 (8.64)			75.76 (8.27)			.13^e^				
	Range	55-120			58-90			—				
	Q1	72			70.5			—				
	Median	78			77			—				
	Q3	83			81			—				
	Missing values, n	6			2			—				
**BMI (kg/m^2^)**												
	Mean (SD)	29.89 (5.30)			31.17 (5.69)			—				
	Range	20-50			21-46			—				
	Q1	26.22			26.14			—				
	Median	28.72			30.86			.21^c^				
	Q3	32.66			34.95			—				
	Missing values, n	26			8			—				
**Smoking (yes), n (%)**												
	Participants, n (%)		13 (6.1)			2 (7.4)			.79^f^			
	Missing values, n		46			12			—			

^a^Not calculated.

^b^Q1: lower quartile.

^c^Calculated using the Mann-Whitney *U* test.

^d^Q3: upper quartile.

^e^Calculated using the independent samples *t* test.

^f^Calculated from crosstabulation using the Pearson chi-square test.

### Questionnaire Results for Chronic Conditions and Medication

Hypertension was the most prevalent comorbidity, and hyperlipidemia was the second most prevalent comorbidity in both the self-acting patient group and combined cooperation and network patient groups. Arthrosis, arthritis, or joint pain was the third comorbidity in both groups. Statistically significant differences between groups appeared in the prevalences of ischemic cardiac disease (*P*=.001), depression or anxiety (*P*=.03), asthma or chronic obstructive pulmonary disease (COPD; *P*<.001), and long-term pain (*P*<.001; Table 5).

In the self-acting patient group, hypertension, hyperlipidemia, joint ailment (arthrosis, arthritis, or joint pain), and long-term pain were also the most prevalent comorbidities assessed separately with each chronic condition (Multimedia Appendix 2).

Metformin was the most common diabetes medication in both the self-acting patient group and the combined cooperation and network patient groups, and there was no difference in the use of metformin between the groups. In the self-acting patient group, a DPP-4 inhibitor was the second most common medication (53/236, 22.5%), while in the combined cooperation and network patient group, the second most common medication was insulin or a biosimilar medication (16/31, 52%). This difference was statistically significant (*P*<.001; Multimedia Appendix 3).

Statistically significant differences were present in the amount of diabetes medication. In the self-acting group, 10.6% (25/235) of the patients did not have any medication for diabetes, 54% (127/235) of the patients used 1 drug, and 18.3% (43/235) of the patients used 2 drugs. In the combined cooperation and network patient group, all patients used diabetes medication, 29% (9/31) of the patients used 1 drug, and 52% (16/31) used 2 drugs (*P*<.001; Multimedia Appendix 3).

ACE inhibitors or AT2 receptor blockers were the most common antihypertensive drugs in both groups. Statistically significant differences between the groups were found in the usage of β-blocker, nitroglycerin, or digitalis medications, which were analyzed as 1 group (*P*<.001); pain medication (*P*=.03); psychopharmacological drugs (*P*=.001); and medication for pulmonary diseases (*P*=.004). The self-acting patients used less of these medications than the patients in the cooperation and network groups (Multimedia Appendix 4).

**Table 5:** Comparison of self-reported chronic conditions in the self-acting group (n=259) and combined cooperation and network groups (n=39) using crosstabulation in the Pearson chi-square test.

Chronic condition	Self-acting group, n (%)	Cooperation and network groups, n (%)	*P* value
Hypertension	189 (83.6)^a^	22 (75.9)^b^	.30
Hyperlipidemia	141 (66.2)^c^	21 (70.0)^d^	.68
Cardiac arrythmia	45 (25.6)^e^	11 (36.7)^d^	.21
Ischemic cardiac disease	31 (17.1)^f^	12 (44.4)^g^	.001
Depression or anxiety^h^	40 (22.5)	12 (41.4)	.03
Gastrointestinal ailment	35 (19.9)^e^	6 (22.2)^g^	.78
Asthma or COPD^h,i^	24 (13.6)	12 (41.4)	<.001
Long-term pain	50 (29.1)^j^	18 (64.3)^k^	<.001
Arthrosis, arthritis, or joint pain^h^	99 (52.4)	19 (67.9)	.13

^a^33 missing values.

^b^7 missing values.

^c^46 missing values.

^d^9 missing values.

^e^83 missing values.

^f^78 missing values.

^g^12 missing values.

^h^2 or 3 conditions were combined in the analysis. Patients responded “yes” if they had 1 or some of these conditions.

^i^COPD: chronic obstructive pulmonary disease.

^j^87 missing values.

^k^11 missing values.

### Questionnaire Results for Self-Rated Health, Disability, Health-Related Quality of Life, and Well-being

Most self-acting patients (130/232, 56%) were satisfied with their health, while 12.9% (30/232) were unsatisfied. In all other groups combined, 53% (18/34) were unsatisfied, and a minority (2/34, 6%) were satisfied. The difference between the self-acting patient group and all other patient groups was statistically significant (Pearson chi-square test, *P*<.001).

The responses of the self-acting patient group and all other patient groups to the WHODAS 2.0 differed by 12 points, which is a 20% difference in the medians, a statistically significant difference (*P*<.001; Table 6).

The difference in medians between the self-acting group and the other groups to the EQ-5D-5L index score values was 0.20 points and to the EQ VAS was 23.82 points, and both differences were statistically significant (*P*<.001). The clinical significance of the differences could be computationally determined with the minimally important difference (MID=0.5 x pooled SD) [33]*.* In our population, the computational MIDs were 0.066 for the EQ-5D-5L index value and 8.48 for the EQ VAS (Table 6).

In addition, statistically significant differences between the groups were detected in the WBQ-12 questionnaire’s dimensions of negative (*P*<.001), positive (*P*<.001), and general well-being (*P*=.004). To assess the clinical significance of the difference in the means for general well-being, we calculated the MID (MID=2.14; Table 6).

**Table 6:** The self-acting patient group’s (n=259) and the other groups’ (combined cooperation, community, and network patient groups; n=45) self-reported disability with World Health Organization Disability Assessment Schedule (WHODAS) 2.0, health-related quality of life using the EQ-5L-5D index value and the EQ visual analog scale (VAS), and health-related well-being (Well-being Questionnaire [WBQ-12]) results.

Variable				Self-acting group		The other groups		*P* value^a^		
**WHODAS 2.0 (range: 12-60)**										
		Mean (SD)	15.91 (4.91)		25.57 (6.72)		—^b^			
		Range	12-36		13-37		—			
		Q1^c^	12.00		19.00		—			
		Median	14.00		26.00		<.001			
		Q3^d^	18.00		31.00		—			
		Missing values, n	42		15		—			
**EQ-5D-5L index value**										
		Mean (SD)	0.831 (0.129)		0.633 (0.153)		—			
		Range	0.295-1.000		0.096-0.859		—			
		Q1	0.755		0.599		—			
		Median	0.856		0.669		<.001			
		Q3	0.903		0.729		—			
		Missing values, n	25		10		—			
**EQ VAS (range: 0-100)**										
		Mean (SD)	77.59 (16.70)		53.77 (18.62)		—			
		Range	10-100		4-90		—			
		Q1	75.00		40.00		—			
		Median	80.00		55.00		<.001			
		Q3	90.00		70.00		—			
		Missing values, n	26		10		—			
**WBQ-12**										
	**Negative well-being (0-12)**									
		Mean (SD)	1.48 (1.73)		2.74 (2.28)		—			
		Range	0-8		0-11		—			
		Q1	0		1.00		—			
		Median	1.00		2.00		<.001			
		Q3	2.00		4.00		—			
		Missing values, n	28		10		—			
	**Energy (0-12)**									
		Mean (SD)	5.27 (1.64)		5.83 (1.62)		—			
		Range	0-10		2-9		—			
		Q1	4.00		5.00		—			
		Median	5.50		6.00		.08			
		Q3	6.00		7.00		—			
		Missing values, n	25		10		—			
	**Positive well-being (0-12)**									
		Mean (SD)	8.02 (2.88)		6.51 (2.23)		—			
		Range	0-12		1-12		—			
		Q1	6.00		5.00		—			
		Median	8.00		6.00		.001			
		Q3	10.00		8.00		—			
		Missing values, n	24		10		—			
	**General well-being (0-36)**									
		Mean (SD)	23.84 (4.23)		21.6 (4.58)		—			
		Range	11-33		7-31		—			
		Q1	21.00		20.00		—			
		Median	24.00		22.00		.004			
		Q3	27.00		24.00		—			
		Missing values, n	28		10		—			

^a^Calculated using the Mann-Whitney *U* test.

^b^Not calculated.

^c^Q1: lower quartile.

^d^Q3: upper quartile.

### Criterion Validity

We explored Navigator’s criterion validity by comparing the nurses’ evaluation of the patient group with the actual result that Navigator proposed. Almost all of Navigator’s results (248/256, 96.9%) for the self-acting patient group were in concordance with the nurses’ evaluations. Two-thirds (67%) of the evaluations were in concordance with the actual Navigator result in the cooperation (24/36) and network (2/3) groups, while the concordance was less than one-third (2/7, 29%) in the community group. One-half (13/26, 50%) of the mismatches were in the evaluation of the patient’s medical state, and 31% (8/26) of the mismatches were in the evaluation of the patient’s functional ability in everyday life. In 19% (5/26) of the mismatching cases, both of these 2 dimensions were evaluated differently (Multimedia Appendix 5).

### Patients’ Ability to Use Electronic Services

Patients responded to Navigator’s question “Do you know how to use electronic services?” on a VAS of 1 (yes) to 10 (no). The self-acting patient group’s median of 1 (Q1=1 and Q3=3; n=259) differed from the other groups’ median of 2 (Q1=1 and Q3=9; n=45). When analyzed with the independent samples Mann-Whitney *U* test, the difference was statistically significant (*P*=.001).

## Discussion

### Principal Findings

Navigator assigned most patients to the self-acting group. The patients in the self-acting group had comorbidities and medication in addition to diabetes but fewer of both than the patients in the cooperation and network groups. The self-acting patients differed from other patient groups in disability, health-related quality of life, most dimensions of well-being, and self-rated health. The proportion of patients over 80 years old was 3 times higher in the other patient groups than in the self-acting group, though the groups’ median ages did not differ significantly. Almost all patients considered their skills at using electronic services to be good. Additionally, the agreement between the professionals’ evaluations of the patient group with the actual result that Navigator proposed (criterion validity) was very high for the self-acting patients.

### Description of the Self-Acting Patient Group

Most self-acting patients were 60 years to 79 years old, and they had had diabetes for less than 10 years. The prevalence of diabetes doubles in the Finnish population at the age of 60 years to 69 years compared with younger age groups [34], and the prevalence peaks in high-income countries in those aged 75 years to 79 years [24], which may reflect our findings regarding the age distribution. The shorter duration of diabetes may impact the self-acting patients’ diabetes care, as they had less diabetes medication, and oral agents were emphasized when compared with the situation of patients in the combined cooperation and network groups. Diabetes care seemed to be well-balanced, as most of the self-acting patients reached all the general targets of diabetes care (HbA_1c_<53 mmol/mol, LDL cholesterol<2.6-2.8 nmol/L, blood pressure<140/80) [35,36]. The differences in HbA_1c_ and LDL cholesterol values between the 2 groups may be associated with the different target values in care. The HbA_1c_ target for older patients rises as their disability reduces [37]. Additionally, the LDL cholesterol target is based on the duration of diabetes (less or more than 10 years), patient’s age, and related comorbidities [35-37].

The self-acting patient group’s most common comorbidities were hypertension, hyperlipidemia, and arthrosis, which is in line with previous findings in multimorbidity research [38,39]. Ischemic cardiac disease, depression or anxiety, and asthma or COPD were more prevalent in the cooperation and network group, which may be associated with the increase in the incidence and prevalence of diabetic complications and multimorbidity in patients of an older age and with a longer history of diabetes [40,41]. These results conform to our findings of the self-acting patients’ medication, as the patients’ smaller number of comorbidities result in a reduced need for medication compared with the patients in the cooperation and network group.

The self-acting patients’ lower number of comorbidities and medication may in turn reflect their better self-rated health, functional ability, health-related quality of life, and well-being, when compared with all other groups.

We noticed a 12-point difference (20% in medians) when assessing disability with the 12-item WHODAS 2.0. A clinically significant difference of 9 points was observed in a Finnish population with chronic musculoskeletal pain [33], as was a change of 5% to 10% in scoring in hospitalized patients [42,43]. Though previous studies were conducted in different settings, we may consider our greater differences clinically significant in favor of the self-acting patients’ better functional ability. This result is also in line with the findings on multimorbidity, defined as the presence of 2 or more chronic conditions, and its association with reduced ability. Functional decline is greater with a higher number of conditions as in the combined cooperation and network patient group, especially when depressive symptoms are included [38,41,44,45].

The differences between the self-acting and all other groups in health-related quality of life measured with the EQ-5D-5L and EQ VAS exceeded the MID values by almost 3 times. When assessing patients with diabetes and chronic pulmonary diseases, the MID in the EQ-5D-5L index score values may vary from 0.03 to 0.069 [46,47] and in the EQ VAS may vary from 0.5 to 9.7 [47,48]. Thus, we may consider our results as clinically significant in favor of the self-acting patients’ better health-related quality of life. Previous findings have shown that patients’ diabetes-related clinical conditions, poor glycemic control, injectable medication, and polypharmacy negatively affect health-related quality of life, which in our results may appear as the other patient groups’ lower values in health-related quality of life [27,49-54].

Health-related well-being was measured with the WBQ-12, and the self-acting patients’ better general well-being score compared with those of the other groups may be clinically significant, as the differences in means between groups again exceeds the MID [33]. In our population, the self-acting patients’ general well-being was a little lower compared with that of patients in other cultures and populations with newly diagnosed type 2 diabetes and those with a greater than 12-year history of the disease [55-57].

Additionally, the results on self-rated health conform with the previous differences between groups in functional ability, health-related quality of life, and general well-being. Aging and multimorbidity have been associated with reduced self-rated health as well, and they presumably impact our findings [26,45,58].

### Patients’ Ability to Use Electronic Services

All patients, not only the self-acting patients, responded that their ability to use electronic services was good. Therefore, advising and empowering all capable patients about utilizing digital equipment and applications in their self-care could improve patient care and care results, release health care resources for patients needing services at face-to-face appointments, and reduce health care costs [1,3-6,8-10]. In addition to the capability, the patients’ willingness, concerns, and fears regarding digital services should be noted, especially with older or otherwise vulnerable patients [12,13,16,17]. Digital alternatives in health services should supplement and support the patient’s individual care instead of complicating and thus harming it.

### Criterion Validity

The concordance of the nurse’s evaluation of the patient’s Navigator result and the actual result was very high for the self-acting patients. The information in the patients’ EHRs and some nurses’ and patients’ relational continuity of care may facilitate patient knowledge and impact the result positively [59,60].

The small number of patients in the other groups complicates the interpretation of the overall criterion validity result. However, the high mismatch percentages in the 3 small groups may indicate that, other than self-acting patients, patients are not easy to identify by a professional. This emphasizes the importance of paying attention to the patients’ views of their values and coping in their life. Previous results concerning Navigator’s user experiences indicated that Navigator’s questions eased raising issues in conversation and helped the professionals to extensively understand the patient’s general care. The questions assisted the patients in understanding their situation better and to see their situation from new perspectives [61]. Thus, the use of Navigator probably impacts the group results, explaining the mismatches. In general, Navigator might be especially useful with patients who do not have notes in EHRs and new patients with newly diagnosed morbidities.

### Limitations

The number of patients to evaluate the patients’ characteristics was sufficient only in the self-acting group. Although the community, cooperation, and networks groups were merged, they still formed a small sample of other than self-acting patients. Significant differences between groups were noted, but individual extreme responses may be emphasized especially in small groups and thus bias the group result. Additionally, some missing values in the questionnaire responses may bias the result. Therefore, our study results of the group differences in medical condition and disability, health-related quality of life, well-being, and self-rated health are intended for generating new hypotheses and need to be confirmed in future studies.

The vast proportion of patients being assigned to the self-acting group may be due to the capability of these patients to attend follow-up appointments at a health center, while older patients may be placed in home health care instead of visiting a health center. Our distribution result may also derive from the patients’ voluntary participation in the study based on their willingness and attitudes toward medical research [62,63] and older and sicker patients’ denial of informed consent, which leads to refusal to participate in a study [64]. This refusal may have reduced the number of patients in the presumed older and sicker patient groups.

This study was performed only at one health center, the study participants were volunteers, and the distribution of this population with diabetes into the self-acting patient group was emphasized. Therefore, this limits the generalizability of our results. Additionally, the differences between groups were noticed particularly between all patients; therefore, at the individual level, a group result for a single patient cannot be determined deductively on the grounds of this study.

In assessing the criterion validity, only 1 nurse evaluated the patient’s incoming Navigator result as in real life. The concordance result may have been different if another professional had conducted the evaluation. The forthcoming reliability study may hone the results, as the Navigator questionnaire was later repeated by the same nurse and also by a physician simultaneously.

### Conclusions

Our results support the hypotheses that the self-acting patients were younger at the group level; had a simpler medical condition; and had better self-rated health, functional ability, health-related quality of life, and well-being. As far as self-acting patients form the largest group of patients with diabetes, their care pathway could be designed to account for their multimorbidity and to take a holistic approach in care integration, emphasizing the approach of preventing diabetic complications and disabilities. Digital services as complementary options for supporting self-care and an alternative to health center visits may be integrated into all care pathways and offered to all patients willing to use them. Efficiently targeting needs-based health services to various patient groups could reduce the worldwide burden and related costs of aging and multimorbid populations in health care. Navigator may add value besides databased patient segmentation methods, as the patients’ own views of their capabilities in everyday life are particularly widely considered with Navigator.

## Appendix

Multimedia Appendix 1 Patients’ distribution into Navigator’s four groups (self-acting, cooperating, community, and network) and the age groups.

Multimedia Appendix 2 The bubble chart presents the self-acting patients’ self-reported comorbidities in addition to diabetes. In the left column, the different conditions are shown and the bubbles show the percentage of those patients responding affirmatively to having the crossing condition. The four major comorbid conditions are hypertension, hyperlipidemia, a joint ailment (arthrosis, arthritis, or joint pain), and long-term pain.

Multimedia Appendix 3 Comparison of DM medication agents and number of DM agents in self-acting group (n=259) and in combined cooperation and network group (n=39).

Multimedia Appendix 4 Comparison of all medication used for chronic conditions in self-acting group (n=259) and in combined cooperation and network group (n=39).

Multimedia Appendix 5 The concordance (c-%) and mismatches (m-%) between the nurse’s evaluation of the patient’s Navigator result before appointments, and the actual result Navigator proposes. The green color indicates the matches, yellow the mismatch in the evaluation of medical state, blue the mismatch in evaluation of functional ability in everyday life, and red the mismatch in the evaluation of both the medical state and functional ability in everyday life (1=self-acting, 2=cooperation, 3=community, 4=network group).

## Abbreviations

COPD: chronic obstructive pulmonary disease
EHR: electronic health record
HbA1c: glycated hemoglobin
LDL: low-density lipoprotein
MID: minimally important difference
UACR: urine albumin to creatinine ratio
VAS: visual analog scale
WBQ-12: Well-being Questionnaire
WHODAS: World Health Organization Disability Assessment Schedule

## Data Availability

The data sets generated and analyzed during the current study are not publicly available due to the privacy policy, but they are available from the corresponding author on reasonable request.
