# Supplementary material for: Segmenting Patients With Diabetes With the Navigator Service in Primary Care and a Description of the Self-Acting Patient Group: Cross-Sectional Study
Source: J Med Internet Res. 2023 Sep 8;25:e40560. doi: 10.2196/40560 (PMC10517389; doi:10.2196/40560)
Supplement: Multimedia Appendix 1 [file jmir_v25i1e40560_app1.docx]

Appendix 1. Patients’ distribution into Navigator’s four groups (self-acting, cooperating, community, and network) and the age groups

|  |  | **Age groups (years), n (% within group)** | | | | |  |  |  |  |
| --- | --- | --- | --- | --- | --- | --- | --- | --- | --- | --- |
|  |  | **≤ 59** | **60 – 69** | **70 – 79** | **≥ 80** | **Total, n (%)** | **Q1ᵃ** | **Md** | **Q3ᵃ** | **SD** |
| **Group, n (%)** | | | | | |  |  |  |  |  |
|  | Self-acting | 32 (12.4%) | 97 (37.5%) | 109 (42.1%) | 21 (8.1%) | 259 (85.2%) | 65 | 70 | 74 | 8.4 |
|  | Cooperating | 6 (17.6%) | 8 (23.5%) | 11 (32.4%) | 9 (26.5%) | 34 (11.2%) | 62 | 73 | 80.25 | 11.6 |
|  | Community | 0 | 3  (50%) | 2 (33.3%) | 1 (16.7%) | 6 (2.0%) | 65.25 | 70 | 78.25 | 8.9 |
|  | Network | 2 (40.0%) | 1 (20.0%) | 1 (20.0%) | 1 (20.0%) | 5 (1.6%) | 49 | 65 | 77.5 | 14.8 |
|  | Total (%) | 40 (13.2%) | 109 (35.9%) | 123 (40.5%) | 32 (10.5%) | 304 (100%) |  |  |  |  |
| *P* value | |  |  |  |  |  |  | *.27ᵇ* |  |  |

ªQ₁ and Q₃ **=** lower and upper quartiles

ᵇ*P* value to compare the groups is calculated using the patient groups' age medians (Kruskal-Wallis test)
