# Supplementary figures and images for: Segmenting Patients With Diabetes With the Navigator Service in Primary Care and a Description of the Self-Acting Patient Group: Cross-Sectional Study
Source: J Med Internet Res. 2023 Sep 8;25:e40560. doi: 10.2196/40560 (PMC10517389; doi:10.2196/40560)

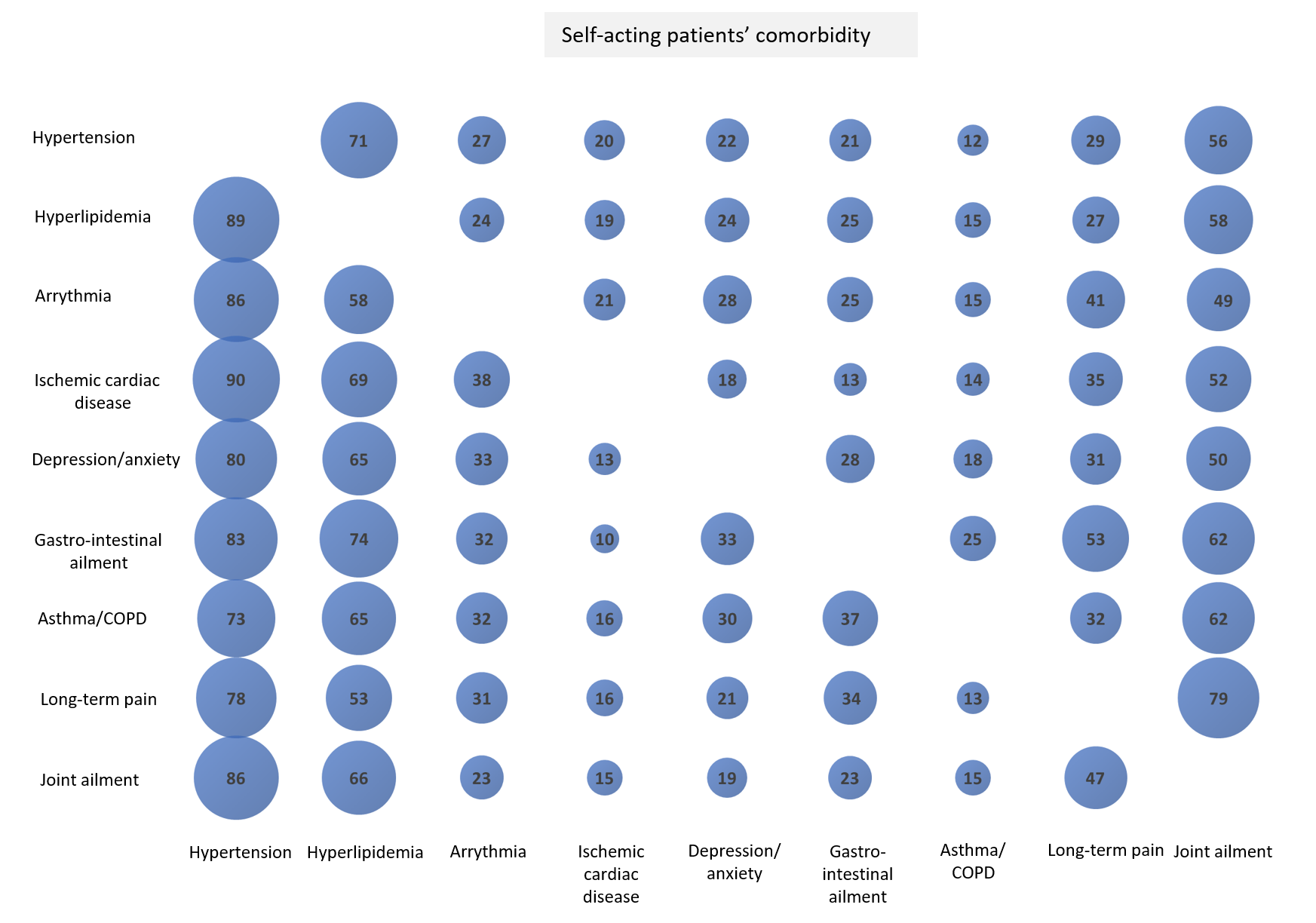

Supplement: Multimedia Appendix 2 [file jmir_v25i1e40560_app2.png]
