# Supplementary material for: Segmenting Patients With Diabetes With the Navigator Service in Primary Care and a Description of the Self-Acting Patient Group: Cross-Sectional Study
Source: J Med Internet Res. 2023 Sep 8;25:e40560. doi: 10.2196/40560 (PMC10517389; doi:10.2196/40560)
Supplement: Multimedia Appendix 3 [file jmir_v25i1e40560_app3.docx]

Appendix 3. Comparison of DM medication agents and number of DM agents in self-acting group (n=259) and in combined cooperation and network group (n=39).

| **Variable** | |  | **Value** | ***P* value^a^** | **Missing** |
| --- | --- | --- | --- | --- | --- |
| **Diabetes medication, n (%)** | | |  |  |  |
|  | Metformin | |  | .919 |  |
|  |  | self-acting group | 180 (76.3%) |  | 23 |
|  |  | cooperation and network group | 24 (77.4%) |  | 8 |
|  | DPP inhibitor | |  | .423 |  |
|  |  | self-acting group | 53 (22.5%) |  | 23 |
|  |  | cooperation and network group | 9 (29.0%) |  | 8 |
|  | SGLT-2 inhibitor | |  | .185 |  |
|  |  | self-acting group | 32 (13.6%) |  | 23 |
|  |  | cooperation and network group | 7 (22.6%) |  | 8 |
|  | Insulin or biosimilar | |  | <.001 |  |
|  |  | self-acting group | 31 (13.1%) |  | 23 |
|  |  | cooperation and network group | 16 (51.6%) |  | 8 |
|  | GLP-1 analog | |  | .492 |  |
|  |  | self-acting group | 15 (6.4%) |  | 23 |
|  |  | cooperation and network group | 3 (9.7%) |  | 8 |
| **Number of diabetes medication, n (%)** | | |  | <.001 |  |
|  | None | |  |  |  |
|  |  | self-acting group | 25 (11.0%) |  | 24 |
|  |  | cooperation and network group | 0 |  | 8 |
|  | One agent | |  |  |  |
|  |  | self-acting group | 127 (54.0%) |  | 24 |
|  |  | cooperation and network group | 9 (29.0%) |  | 8 |
|  | Two agents | |  |  |  |
|  |  | self-acting group | 43 (18.3%) |  | 24 |
|  |  | cooperation and network group | 16 (51.6%) |  | 8 |
|  | Three to four agents | |  |  |  |
|  |  | self-acting group | 32 (13.2%) |  | 24 |
|  |  | cooperation and network group | 6 (19.4 %) |  | 8 |

^a^*P* value from crosstabulation (Pearson’s Chi-square test)
