# Supplementary material for: Segmenting Patients With Diabetes With the Navigator Service in Primary Care and a Description of the Self-Acting Patient Group: Cross-Sectional Study
Source: J Med Internet Res. 2023 Sep 8;25:e40560. doi: 10.2196/40560 (PMC10517389; doi:10.2196/40560)
Supplement: Multimedia Appendix 4 [file jmir_v25i1e40560_app4.docx]

Appendix 4. Comparison of all medication used for chronic conditions in self-acting group (n=259) and in combined cooperation and network group (n=39).

| **Variable** | | | **Value for**  **one agent** | **Value for two**  **or more agents** | ***P* value^a^** | **Missing** |
| --- | --- | --- | --- | --- | --- | --- |
|  | **ACE or AT2 (±HCT), n (%)** | |  |  | .423 |  |
|  |  | self-acting group | 169 (71.9%) | - |  | 24 |
|  |  | cooperation and network group | 20 (64.5%) | - |  | 8 |
|  | **β-blocker /nitroglycerin / digitalis** | |  |  | <.001 |  |
|  |  | self-acting group | 92 (39.1%) | 15 (6.4%) |  | 24 |
|  |  | cooperation and network group | 7 (22.6%) | 8 (25.8%) |  | 8 |
|  | **Calcium-channel blocker / diuretic / other antihypertensive** | |  |  | .067 |  |
|  |  | self-acting group | 73 (31.1%) | 21 (8.9%) |  | 24 |
|  |  | cooperation and network group | 8 (25.8%) | 7 (22.6%) |  | 8 |
|  | **Statin / other antihyperlipidemic** | |  |  | .586 |  |
|  |  | self-acting group | 158 (67.2%) | 6 (2.6%) |  | 24 |
|  |  | cooperation and network group | 20 (64.5%) | 0 |  | 8 |
|  | **Pain medication** | |  |  | <.033 |  |
|  |  | self-acting group | 50 (21.3%) | 25 (10.6%) |  | 24 |
|  |  | cooperation and network group | 10 (32.3%) | 7 (22.6%) |  | 8 |
|  | **Psychopharmacological agent** | |  |  | <.001 |  |
|  |  | self-acting group | 32 (13.6%) | 16 (6.8%) |  | 24 |
|  |  | cooperation and network group | 6 (19.4%) | 8 (25.8%) |  | 8 |
|  | **Pulmonary disease medication** | |  |  | .004 |  |
|  |  | self-acting group | 10 (4.3%) | 12 (5.1%) |  |  |
|  |  | cooperation and network group | 3 (9.7%) | 6 (19.4%) |  |  |

^a^*P* value from crosstabulation (Pearson’s Chi-square test)
