# Supplementary material for: Segmenting Patients With Diabetes With the Navigator Service in Primary Care and a Description of the Self-Acting Patient Group: Cross-Sectional Study
Source: J Med Internet Res. 2023 Sep 8;25:e40560. doi: 10.2196/40560 (PMC10517389; doi:10.2196/40560)
Supplement: Multimedia Appendix 5 [file jmir_v25i1e40560_app5.docx]

**Appendix 5.** The concordance (c-%) and mismatches (m-%) between the nurse’s evaluation of the patient’s Navigator result before appointments, and the actual result Navigator proposes. The green color indicates the matches, yellow the mismatch in the evaluation of medical state, blue the mismatch in evaluation of functional ability in everyday life, and red the mismatch in the evaluation of both the medical state and functional ability in everyday life (1=self-acting, 2=cooperation, 3=community, 4=network group).

| **The Navigator service’s results** | | | | | | | | |
| --- | --- | --- | --- | --- | --- | --- | --- | --- |
|  |  | | **1** | **2** | **3** | **4** | **Total** | **c-%** |
| **Nurse’s evaluation of**  **patient’s Navigator result** | | **1** | **248** | 5 | 3 | 0 | 256 | 96.9% |
|  |  | **2** | 8 | **24** | 1 | 3 | 36 | 66.7% |
|  |  | **3** | 1 | 4 | **2** | 0 | 7 | 28.6% |
|  |  | **4** | 0 | 1 | 0 | **2** | 3 | 66.7% |
|  | | **Missing** | 2 | 0 | 0 | 0 | 2 |  |
|  | | **Total** | 259 | 34 | 6 | 5 | 304 |  |
|  | | **m-%** | 3.5% | 29.4% | 66.7% | 60.0% |  |  |
